# Supplementary material for: Accumulation Bias in meta-analysis: the need to consider time in error control
Source: F1000Res. 2019 Jun 25;8:962. [Version 1] doi: 10.12688/f1000research.19375.1 (PMC6808047; doi:10.12688/f1000research.19375.1)
Supplement: Peer review history

# Peer review: Reviewer response for version 1

**DOI:** 10.5256/f1000research.21241.r53065

**Published:** 2019-10-16

**Authors:** Joanna IntHout

**Affiliations:** 1 Department for Health Evidence, Radboud University Medical Center, Nijmegen, The Netherlands

The paper explains how bias arises in meta-analysis, as studies never are a random sample and the timing of a meta-analysis neither is random. Timing of the meta-analysis – if performed – and the results of the studies are obviously dependent, resulting in accumulation bias and inflated type I errors. The parallel between conducting studies and betting while continuously reinvesting the profits is an intriguing one.

 I apologize for the long duration of my peer review: the paper was rather intense and long, and sometimes a bit difficult to follow, due to the condensed writing style. It might benefit from some extra sentences and some additional, concrete examples. And, to my opinion the content might also be suitable for a few papers.

 However, I could find only minor editorial issues, and I congratulate the authors with this well-written, very relevant paper.

Page 1, abstract

 It feels a bit like a contradiction when is stated (halfway):  “…, no valid p-value test can be constructed. Second, tests based on likelihood ratios withstand Acc. Bias: they provide bounds on error probabilities that remain valid despite the bias.” Also last paragraph: Taking up likelihood ratios… allows for valid tests.

 Probably it has to do with the term p-value based statistical tests that I am not familiar with (as opposed to likelihood ratio tests). I think it has to do with the explanation in the discussion, that (p-value) tail area approaches to testing are very sensitive to the shape of the sampling distribution, but in the abstract, this was not clear to me.

1. Introduction

 I like how you start with the two quotes.

 First column at the end: knowledge and all decision **S** made along the way. (s is missing)

2. Accumulation Bias

 Somewhat difficult (although correct) sentence: The crucial point is that not all pilot studies or small study series will reach a meaningful size and that doing so might depend on results in the series.

 One but last sentence: So meta-analysis also report….  (should be meta-analys **e**s)

Section 3.6

 Typo: The inflation actual inflation in the type-I error…

 Figure 1: in black and white print the colours in the legend seem to differ from the colours in the graph.

 Table 2: If possible, it might be handy to add the P̃ _0_ and P _0_ to the title (after bias only and after bias as well as impaired sampling distribution).

Section 4.5

 Typo: These cumulative meta-analysis judge…. : should be meta-analys **e**s

Section 5

 Here you refer to “another illustration”… as the *Toy Story* Scenario. However, where do you discuss this scenario?

Section 6.1

 Typo: “But this effects…”. Also somewhat unclear sentence. I suppose that you mean that is difficult to define a cut-off for the number of early studies to be excluded from meta-analysis.

Section 6.2

 Here you compare the prior odds of Ioannidis with the fraction of pilot studies from the null and alternative distribution π / (1-π). However, you did not define π before, and if I understand it correctly, π is the fraction of studies from the alternative distribution, although this text (first line) suggests the other way around.

Section 7.1

 Formula 7.2: Please define H _1_ and H _0_.

 Please edit sentence directly below formula: …16 times as many true rejections than (as?) false rejections (with?) γ = (1-β) / α.

 Formula 7.4: P _0_ : should be integral over z _1_, …, z _t_ (instead of z _2_) (twice). Should it not contain also dz(1).…dz(t)?

 I noticed that you don’t use the word test, only “error control”. It is not fully clear to me: if we use the threshold based on the Bayes posterior odds, does that also result in a p-value, or is it just a yes/no answer? Or can we use a distribution? (you elaborate on this only in Section 9).

 And how do we specify R= π / (1-π)?  Should this be influenced by the study results seen thus far? As you state in section 6.2 on π / (1-π): “the fraction of pilot studies from the null and alternative distribution. … The only available source of information would be previous study results… “. However, this would mean that – indeed -  depending on the timing of the meta-analysis, we would define a different R.

 Or should we use the same – more general -  R as Ioannidis, ie 1:1, or 2:1?

 Interesting is also that the threshold, that is based on the pre-experimental rejection odds, becomes more stringent if we believe the ratio of true to false rejections to be higher, e.g. if R =2 and γ = 16, the threshold becomes 32, but if R=1, the threshold is 16. Could you elaborate on that? You do elaborate a bit in Section 8, but for me it is still not very clear.

Section 9

 Edit, 2 ^nd^ paragraph, … a series of bets s(Z _1_), … against the null hypothesis that make a profit….

 I suggest to (re)move “against the null hypothesis”  to facilitate easier reading.

 Typo: each null result might cost **s** 1 dollar (should be: cost)

 “If you cash out, your profit is s(Z _1_) s(Z _2_) s(Z _3_)”. Are the s(Z) bets not odds or probabilities?  Should we not add the profit here? And subtract the 1 dollar initial investment? Or do I show here my lack of knowledge on gambling?  Only later, when you suggest s(Z) = f _1_/f _0_, this makes more sense.

 Typo, second column before quote of Shafer: twice “under the null”.

 Inspecting the betting profit of a study: do you mean calculating the LR for that study?

 I don’t understand the following sentence: “The LR has the ability to maximize the rate of growth among all studies in a series”.

Section 10

 Typo: 3 ^rd^ paragraph: separate meta-analysis of various sizes…, should be meta-analys **e**s

Is the work clearly and accurately presented and does it cite the current literature?

Partly

If applicable, is the statistical analysis and its interpretation appropriate?

Yes

Are all the source data underlying the results available to ensure full reproducibility?

Yes

Is the study design appropriate and is the work technically sound?

Yes

Are the conclusions drawn adequately supported by the results?

Yes

Are sufficient details of methods and analysis provided to allow replication by others?

Yes

Reviewer Expertise:

Biostatistics (i.e. statistician in medical field), with an emphasis on meta-analysis

I confirm that I have read this submission and believe that I have an appropriate level of expertise to confirm that it is of an acceptable scientific standard.

# Peer review: Reviewer response for version 1

**DOI:** 10.5256/f1000research.21241.r50370

**Published:** 2019-08-14

**Authors:** Steven P. Ellis

**Affiliations:** 1 Department of Psychiatry, New York State Psychiatric Institute (NYSPI), Columbia University, New York City, NY, USA

The article explains how bias arises in meta-analysis and explains how, in theory, one can nonetheless control the error rate through use of the likelihood ratio statistic. I found the use of the likelihood ratio quite interesting.

 2.2, p. 4 "This is also the case when the timing of the meta-analysis is based on an (overly) optimistic last study estimate or an (overly) optimistic meta-analysis synthesis is considered the final one."

 Section 3, p. 4. "We denote this example ..." How about "We CALL this example ..."

 (3.1a), p. 5: I think it should just be *n _i_*, not √ *n _i_*. [Maybe not?]

 p. 5: "... if the study shows a significant positive effect" So you are doing one-sided tests. Why do you do alpha/2-size tests, for example 0.025? Why not alpha-size, for example 0.05? Getting a significantly low Z can have the interpretation that that treatment being tested seems to actually be harmful. Therefore no further studies should be done with it.

 p. 5: Why is *T *≥ *t *+ 1? Why might the current study ultimately prove to be the last one?

 p. 6: It seems that the P in (3.2) has nothing to do with the null and alternative hypotheses. That P has to do with the behavior of researchers.

 p. 6: The rate 2.5/(2.5+1.9) might be justified by observing that that number is just the conditional probability of getting a positive finding conditional on another study being done.

 p. 6: "As a result, study series that contain more significant studies have larger probabilities to come into existence than those that contain less." That sentence is vague.

 p. 6: There appears to be a typographical error in formula (3.3). The factors alpha/2 and (1-alpha) shouldn't be in the numerator. However, the value 0.487 on the right-hand side is correct. (Checked by simulation.)

 p. 6: "... the last study is unbiased ..." What do you mean by "last study", the Tth study? The last study before what? Let S denote the number of studies available at the time of the meta-analysis. S is random. But the Sth study is not unbiased (given that there will be a meta-analysis) because the decision to do the meta-analysis partly depends on the outcome of study S. Since the subject of the paper is meta-analysis, it is the first S studies, i.e., the studies available at the time of the meta-analysis, that are relevant.

 Suppose U is a fixed or random time that is statistically independent of the study series. Is the last study before U unbiased? What if the last study before U was published 50 years ago? The fact that no study has been done in 50 years probably says something about the outcome of the last study. So even though U is independent of the study series, conditional on the event that no study has been done in the last 50 years prior to U, that last study is not unbiased. (Perhaps one should pay attention not just to the number of studies that have been performed but also to when they were performed.) It is very difficult to identify a study that is unbiased conditional on everything one knows about the study series. Instead of looking at past studies, one could look at a future study. One might say "I will start a meta-analysis after the next study is completed". The next study would be unbiased, but what if there are no further studies? That strategy would work if one knew for sure that there will be a study. For example, if you knew at time U that some researchers have already started -- but not completed -- a study.

 p. 7: "inflation actual inflation" looks like a typo.

 p. 7: Equation (3.5). Have you defined the P̃ _0_ notation? I think you should one sided-tests. (See above.)

 p. 8: Probability Notation is a conditional probability. The notation “ *z*_1_,…, *_Zt_*” for a study series apparently hasn't been introduced yet.

 p. 9: I don't understand the footnote.

 P. 10: I don't understand the sentence "In this section we assume that the timing of the meta-analysis test is independent from the estimates that determined the size of the series."

 p. 15: "But this effects will be ..." Perhaps this should be "But THESE effects will be ...".

 p. 16: "... which has the same meaning as the fraction ..." Perhaps this should be "... which has the same meaning as the RATIO ..."

 P. 16: "The likelihood ratio is a test statistic that depends on the specification ..." Perhaps this should be "The likelihood ratio is a test statistic that ONLY depends on the specification ..."

 p. 16: "Any data sampled from an alternative distribution will have the same analysis time probabilities as data ..." I prefer "GIVEN THE DATA, any data sampled from an alternative distribution will have the same analysis time probabilities as data ..."

 p. 17: The authors sometimes introduce symbols without defining them. For example, I couldn't find any place where the symbol γ is defined. From context one can figure out what it means, but I would prefer if it were defined somewhere.

 p. 19: "In contrast to an all-or-nothing test for one study, inspecting the betting profit of a study is a way to test the data without loosing the ability..." I think it should be "In contrast to an all-or-nothing test for one study, inspecting the betting profit of a study is a way to test the data without LOSING the ability...".

Is the work clearly and accurately presented and does it cite the current literature?

Yes

If applicable, is the statistical analysis and its interpretation appropriate?

Yes

Are all the source data underlying the results available to ensure full reproducibility?

No source data required

Is the study design appropriate and is the work technically sound?

Yes

Are the conclusions drawn adequately supported by the results?

Yes

Are sufficient details of methods and analysis provided to allow replication by others?

Yes

Reviewer Expertise:

multivariate analysis, statistical computing, applications of topology to data analysis

I confirm that I have read this submission and believe that I have an appropriate level of expertise to confirm that it is of an acceptable scientific standard.
